# The Nordic Maintenance Care Program – An interview study on the use of maintenance care in a selected group of Danish chiropractors

**DOI:** 10.1186/1746-1340-17-5

**Published:** 2009-06-17

**Authors:** Lars Top Møller, Michael Hansen, Charlotte Leboeuf-Yde

**Affiliations:** 1 Private practice, Grenaa, Denmark; 2 Private practice, Haderslev, Denmark; 3 Research Unit for Clinical Biomechanics, University of Southern Denmark, Backcenter Ringe, Lindevej 5, DK-5750 Ringe, Denmark

## Abstract

**Background:**

Although maintenance care appears to be relatively commonly used among chiropractors, the indications for its use are incompletely understood. A questionnaire survey was recently carried out among Swedish chiropractors in order to identify their choice of various management strategies, including maintenance care. That study revealed a common pattern of choice of strategies. However, it would be necessary to verify these findings in another study population and to obtain some additional information best collected through an interview.

**Objectives:**

The main aim of the present study was to attempt to reproduce the findings in the Swedish study and to obtain more information on the use of maintenance care.

**Method:**

A group of 11 chiropractors were selected because they used maintenance care. They were interviewed using the questionnaire from the previous Swedish survey. The questionnaire consisted of a simple description of a hypothetical patient with low back pain and nine possible ways in which the case could develop ("scenarios"). They could choose between six different management strategies for each scenario. In addition, the chiropractors were encouraged to provide their own definition of maintenance care in an open-ended question. Interviews were taped, transcribed and analyzed. For the open-ended question, statements were identified relating to six pre hoc defined topics on the inclusion criteria/rationale for maintenance care, the frequency of treatments, and the duration of the maintenance care program.

**Results:**

The open-ended question revealed that in patients with low back pain, maintenance care appears to be offered to prevent new events. The rationale was to obtain optimal spinal function. There appears to be no common convention on the frequency of treatments and duration of the treatment program was not mentioned by any of the interviewees.

**Conclusion:**

The results from the questionnaire in the Danish survey showed that the response pattern for the nine scenarios was similar to that obtained in the Swedish survey. There seems to be relative agreement between chiropractors working in different countries and sampled through different methods in relation to their choice of management strategies in patients with low back pain. However, more precise information is needed on the indications for maintenance care and its treatment program, before proceeding to studying its clinical validity.

## Introduction

Secondary and tertiary prevention (so-called maintenance care) appears to be a relatively common clinical management strategy among some chiropractors. Although it appears to be a reasonable approach in patients with recurring back problems, an overzealous use of prolonged treatment programs would threaten the credibility of the chiropractic profession.

The scientific literature on this subject is sparse. Therefore, the use of maintenance care is not based on evidence. For example, its exact indications have not been clarified nor is it known if maintenance care has the intended effect. Only one study on treatment effect has been published; a pilot study on patients with low back pain but with inconclusive results [1]. Several authors have surveyed the chiropractor profession with the intent of learning more about maintenance care, but most study samples were probably unrepresentative of the chiropractic profession and therefore of little use [2,3].

Obviously, this is an important subject that deserves serious study. The ultimate goal would be to establish if maintenance care is clinically useful, and if so, for which type of patients and which type of approach. A literature review, published in 1996, concluded that it would be necessary to perform a series of preliminary studies to delineate the parameters of care, *before *being able to conduct a large-scale multi-center clinical trial [2]. A recent review concluded that scientific status of maintenance care was largely unchanged [3]. For that reason the need persists to perform a number of preliminary studies.

Therefore, before embarking on such a largely unstudied method of treatment, it would be necessary to find out, first, when it is commonly used in daily clinical practice, and when it is considered definitely unsuitable. For this purpose, a number of studies were carried out. For example, a questionnaire survey was recently conducted among a selected group of Swedish chiropractors, in which a basic case was described of a patient with low back pain who had undergone an initial period of chiropractic care [4]. The participants were asked to choose between several follow-up management strategies. These strategies went from referring the patient out for a second opinion to advising the patient to remain in a maintenance care program. The results revealed that there was fair agreement among the participants on the choice of management strategies, notably for scenarios that indicated the need for clinical vigilance.

However, this survey did not provide any information as to whether chiropractors also consider primary prevention as a form of maintenance care. It was also unclear if the response pattern would be similar in different study populations.

We therefore decided to approach the same subject in different study populations. Consequently, two additional studies were performed; one in Denmark and the second in Finland. This report deals with the Danish study, which consisted of an interview with a small group of chiropractors, specifically selected because they used maintenance care in their practice. They were chosen to ensure that we would get informed answers on maintenance care. By collecting the information in this study through an interview, we hoped also to be able to capture some more subtle points, which might have gone undetected in the previous questionnaire study, and it made it possible to solicit the chiropractors' own definitions of maintenance care.

## Method

### Procedure

Eleven Danish chiropractors were selected by a senior chiropractor with knowledge of their clinical practice profile. The inclusion criteria were that they used maintenance care and were willing to submit to the interview. The interview instrument (described below) was sent out per mail and replied to in a subsequent telephone interview, conducted by the first two authors, who also analyzed the data.

### The structured interview

The same questionnaire as that used in the previous Swedish survey was employed [3]. It had been designed by the group of Swedish chiropractors, who were responsible for the first survey, and further refined in a series of small pilot tests, in which the intellectual quality and clarity of the questions was addressed. The questionnaire that was written in English consisted of a basic case, describing a hypothetical patient with low back pain.

The basic case was described as follows: *"A 40-year old man who consults you for low back pain with no additional spinal or musculoskeletal problems, and with no other health problems. There are no aggravating factors at work or at home. His X-rays are normal for his age. There are no "red flags"."*

This case was followed by a description of nine possible ways in which this case could develop ("scenarios") (Additional file 1). For each scenario the respondents could choose between six different clinical management strategies in response to the question: "What would you recommend?" In the questionnaire, these are listed under A-F. An additional, "neither of the above, please explain"- alternative was also available (G), with the possibility to provide ones own alternative answer (Additional file 2).

Replies to the questionnaire were noted by the interviewers as categorical answers, i.e. strategy A, B, or C etc. Suggested additional strategies were noted down. If a new suggested alternative (G) was, in fact, identical with one of the previously suggested alternatives, it was placed under the correct alternative. Combinations of replies were coded as "several replies" and only truly original suggestions could be left under alternative "G", i.e. "None of the above, please explain".

The interpretation of all replies was done independently by the first two authors. Answers on which both agreed were considered factual. In cases of disagreement, the third author would be called in.

The replies to each question were reported as numbers and percentages and compared with the previous results obtained in the Swedish study. For ease of comparison, results from the Swedish study are included in the Table 1. The most commonly selected management strategy was identified for each scenario. To make the report more reader-friendly, the first six of these management strategies have been described also in brief, colloquial terms, as shown in Additional file 2.

**Table 1:** How 11 Danish chiropractors, who use maintenance care, would choose their continued case management strategies in nine hypothetical case scenarios for a patient with low back pain

**The nine case scenarios**	**Strategy A** **"2^nd ^opinion"**	**Strategy B** **"Quick-fix"**	**Strategy C** **"Try again"**	**Strategy D** **"Ext. help – keep in touch"**	**Strategy E** **"Symptom-guided MC"**	**Strategy F** **"Clinical findings-guided MC"**	**Several replies**
**1.**							
**DK**	0	**55**	18	0	9	18	0
**S**	0	**54**	2	0	20	19	2

**2.**							
**DK**	0	27	18	0	**36**	18	0
**S**	0	14	3	0	**44**	30	2

**3.**							
**DK**	0	0	**64**	18	9	0	9
**S**	20	0	**42**	12	8	10	0

**4.**							
**DK**	0	0	0	0	**72**	28	0
**S**	0	7	3	3	**46**	34	0

**5.**							
**DK**	9	0	**36**	9	27	18	0
**S**	14	0	**25**	17	10	24	2

**6.**							
**DK**	0	0	**55**	27	18	0	0
**S**	5	0	**37**	29	7	10	2

**7.**							
**DK**	0	0	**55**	9	18	18	0
**S**	15	2	**32**	15	12	8	2

**8.**							
**DK**	**55**	0	27	0	0	0	18
**S**	**59**	0	14	14	0	0	2

**9.**							
**DK**	**45**	0	18	27	0	0	9
**S**	**59**	0	8	19	0	0	2

Descriptions of the nine case scenarios (1–9) and the six main management strategies (A-F) are found in Additional files 1 and 2. The estimates from a previous Swedish survey (N = 59) have been incorporated in the table, listed below those from the Danish interview. The results are reported as percentages and the largest estimate for each case scenario has been highlighted.

DK = Results from present Danish interview

S = Results from previous Swedish survey (**3**)

MC = Maintenance care

### The open-ended question: "What is your definition of the concept of maintenance care?"

In addition, the chiropractors were encouraged to provide their own definition of maintenance care as response to an open-ended question ("What is your definition of the concept of maintenance care?"). These interviews were taped, transcribed and analyzed. Already during the interviews it became clear that the chiropractors generally failed to provide a clear definition of maintenance care but that they rather described various aspects associated with it. For this reason, we had to approach the analysis in a different way by specifically looking for statements that related to 6 pre hoc defined headings relating to: 1) LBP history, 2) type of symptoms, 3) severity of symptoms, 4) specific objective findings, 5) frequency of treatments, and 6) duration of maintenance care program. Statements that did not fit under any of these headings would be listed separately with the intent of finding additional descriptors under which they could be classified. Explanations relating to the definition of maintenance care were thereafter placed under the relevant pre- and post hoc defined headings. This analysis was done independently by the first two authors. Items on which both agreed were considered factual. Cases of disagreement would be discussed with the third author. Finally, the frequency of statements under each heading was quantified and entered into a table.

### Proportion of patients treated with maintenance care

At the interview, the participants were asked to identify the number of patients receiving maintenance care out of their total number of patient visits, on the day of the interview. From this information it was possible to calculate the percentage of maintenance care patients seen by each chiropractor, and the mean and median values for the whole group.

### Ethics

The participants were known to the first two authors, and the interviews were recorded, later to be transcribed, with the permission of the interviewees. To protect their anonymity, no names were included in the transcribed document and tapes were destroyed as soon as the written text was available thus cutting the link between the participants and their responses. No permission from the local ethics committee is needed for this type of project.

## Results

The eleven interviewed chiropractors reported having had a mean of 35% and median of 42% of maintenance care patients on the day of the survey, ranging from 0% to 70%. The chiropractor with 0% stated that he/she, exceptionally, did not have any such patients on that day but would normally see patients under maintenance care programs.

### The use of maintenance care according to the structured interview – results of the Danish study

No problems in understanding or relating to the questions of the survey were noted among the respondents. Also the analysis was unproblematic.

As can be seen in Table 1, maintenance care would be recommended by at least some of the respondents for all but the last two scenarios. Both symptom-guided and clinical findings-guided maintenance care were noted for the remaining scenarios.

Some type of maintenance care was the most common answer for scenarios 2, 4 and 5. The largest degree of consensus was noted for scenario 4, which described quick and full recovery in a patient with recurrent symptoms. "Symptom-guided maintenance care" was the preferred answer for 72% whereas the remaining 28% would recommend "clinical findings-guided maintenance care", i.e. 100% would recommend maintenance care.

In all 54% would recommend some type of maintenance care for scenario 2; a worried patient who asks for maintenance care but who had only 2 days' of LBP and no previous history. For scenario 5, which is described as a patient with several short-lasting events over the past year but with no obvious improvement after 6 visits, some type of maintenance care was also considered relevant by 55% of the respondents. However, the single most common response for scenario 5 was "try again".

On the other hand, none of the participants suggested any form of maintenance care for scenario 8 (patient getting gradually worse) or scenario 9 (fluctuating symptoms with signs of co-morbidity). Instead, the largest groups (55% and 45%, respectively) agreed that such patients should be submitted to a "second opinion".

More than half of the group agreed on the "quick fix" approach in scenario 1 (immediate recovery and no previous low back pain).

Almost 2/3 would "try again" with the patient in scenario 3 (unexpected lack of improvement in seemingly uncomplicated case). "Try again" was the preferred management strategy also in scenario 6 (initial substantial improvement followed by stagnation), and in scenario 7 (initial substantial improvement followed by gradual worsening).

Strategy D ("external help – keep in touch") or strategy G ("other") were never the most often selected responses.

### Open-ended question: "What is your definition of the concept of maintenance care?"

Only one of the chiropractors expressively defined maintenance care, using the terms of primary, secondary and tertiary prevention, stating that he/she would use maintenance care synonymously with secondary and tertiary prevention only, and. not as a means to prevent the incidence of low back pain (primary prevention).

The other chiropractors preferred to describe the typical maintenance care patient and how this patient would be managed. As can be seen in Table 2, answers were found that fitted four of the six pre-defined headings (1) history of low back pain, 2) type of symptoms, 3) severity of symptoms, and 6) frequency of treatments. None mentioned 4) specific objective findings or 6) duration of maintenance care program. One extra heading was identified: 7) aim of the maintenance care program. There were no problems associated with placing statements under the relevant headings.

**Table 2:** Results from the open-ended question: "*What is your definition of the concept of maintenance care?" *Interview of 11 Danish chiropractors who use maintenance care

**Type of information**	**Description of responses**
1. History of low back pain	*Three *chiropractors mentioned the patient's history, stating that maintenance care was suitable for patients who had recurrent symptoms over a long period.

2. Type of symptoms	*Seven *of the chiropractors mentioned or implied that maintenance care patients do not necessarily have to have any symptoms, when coming to the clinic for a "check up".

3. Severity of symptoms	*Six *of the chiropractors mentioned that maintenance care can be offered regardless the severity of symptoms.

4. Specific objective findings	-

5. Frequency of visits	*Five *of the respondents mentioned the frequency with which they would schedule visits for their maintenance care patients. The total range was between 1 and 6 months and three mentioned an interval of 3 months. They also mentioned that the timing of appointments would vary with the needs of the patient.

6. Duration of maintenance care program	-

7. Aim of the maintenance care program	*Ten *of the eleven chiropractors agreed that the main purpose of maintenance care is to check up on the patient. They *all *intended to help the patient improve spinal function, hereby avoiding a relapse of symptoms.

In order of the frequency with which the information was provided, maintenance care can be described as follows: The aim is to prevent relapses by improving spinal function, and the consultation is a "check-up". Thus, it is irrelevant whether the patient has symptoms or not and the severity of symptoms is also not important. The frequency with which the patient is scheduled would vary with the needs of the patient, probably between 1 and 6 months, with 3 months being relatively common. An indication for maintenance care would be recurrent symptoms over a long period of time.

## Discussion

This was a study of a small group of Danish chiropractors and their theoretical approach to the long-term management of low back pain. They could choose between 6 management strategies for 9 different scenarios. Two of these strategies consisted of different types of maintenance care. We noted that there was a fair amount of concordance in their choices of strategies.

The participants in the Danish study had been selected because they were known to use maintenance care, so we would have expected them to have a higher usage of maintenance care, as compared to a larger group of Swedish chiropractors, who had been selected on other inclusion criteria for participation in a previous questionnaire survey [4]. This was true, as their median number of maintenance care patients was about twice as high as that of the Swedish participants (42% vs. 20%).

Despite this difference, the overall results of the present study mirrored those of the previous Swedish study because, in both study groups, the majority of participants selected the same management strategies. For example, the two groups largely agreed on when to refer patients for a second opinion, when to consider the treatment completed, when to continue the treatment a few times more, and when maintenance care would be their strategy of choice. Hence maintenance care was not more often the predominant answer for the Danish than for the Swedish respondents. Nor was clinical findings-guided maintenance care generally more commonly selected in the Danish than in the Swedish study. Although we could find no specific information in other studies on this topic, this could indicate that the chiropractic clinical approach to this type of patient is fairly universal.

As in the Swedish study, maintenance care was never thought to be relevant for patients who obviously should be referred out for a second opinion. It would therefore appear that also these chiropractors have a safe approach to the long-term management.

The strongest support (100%) for maintenance care was found for the fourth scenario, a patient with recurring back problems and good recovery. On condition that one believes in the usefulness of this approach, for us, we considered this case to be the most suitable indication for maintenance care.

However, there was one response that surprised us. Although the most common single answer to scenario 5 (a patient who failed to improve after six visits) was to "try again" (45%), there was an equally strong preference for some sort of maintenance care. We would have expected that improvement would have been a prerequisite for maintenance care. Also in the Swedish survey the combined result for the two types of maintenance care exceeded the most commonly selected choice ("try again"). We therefore conclude that it would be necessary to study more closely the short-term outcome criteria for patients considered suitable for maintenance care.

We were curious to see, if the use of clinical findings-guided maintenance care would be more commonly considered in this group of practitioners than in the more diversified group surveyed in Sweden. In other words, would these chiropractors ignore patients' symptoms and base their treatment on clinical findings only, such as palpation? Interestingly, this was the case in only 1 of the 9 scenarios. It therefore looks as if the group of Danish chiropractors took notice of their patients' symptoms and not only their own examination findings.

### Interview vs. mail survey – validation of the questionnaire

In a short questionnaire, it can be difficult to convey meaningful clinical information to be understood by a wide variety of individuals. By using the same questionnaire as in the Swedish study but collecting the information through a structured interview, it would be possible to pick up any misunderstandings or comments that could shed light on problems of interpretation in the original questionnaire. Fortunately, the interview revealed no difficulties for the chiropractors to understand or relate to the questionnaire.

### Open-ended question-results

The present study included also an open-ended question that was meant to shed some more light on the definition of maintenance care. However, with one exception, the chiropractors in the present study did not provide distinct definitions of maintenance care but approached the question indirectly, by describing some clinical aspects thereof.

In doing so, they did not describe any conditions or profiles or any standard management programs. Rather, their responses indicated that maintenance care can be used for any type of low back pain with the intent of improving spinal function in order to avoid a relapse of the condition. In fact, this corresponds to the definition of secondary prevention, and none of the respondents discussed a tertiary prevention scenario.

What people think that they do and what really happens, do not always concur. According to a recent Norwegian multi-center outcome study of patients with more persistent or chronic low back pain, at the one-year follow-up, maintenance care was shown to have been given mainly to patients who did *not *have a good short-term outcome [5]. In other words, absence of symptoms in the short term did not seem to incite further treatment (secondary prevention) but continued symptoms did (i.e. tertiary prevention). Whether this was the chiropractors' preferred choice or whether patients with quick recovery were uninterested in secondary prevention, is of course not known.

Some of the interviewed chiropractors considered it irrelevant, if the patient had symptoms or not, at the time of the consultation ("clinical-findings guided maintenance care"). If patients' symptoms are thought to be irrelevant, the chiropractor would have to trust his own examination findings entirely. This is a big responsibility, in the absence of evidence for the validity of the tests used to determine the presence of the treatable spinal lesion or without much knowledge of other factors that precipitate the recurrence of low back pain. Obviously, this concept requires further study.

Further, almost all of the interviewees were of the opinion that chiropractic treatment can improve spinal function and that optimal spinal function will reduce the risk for recurring problems of low back pain. This may be a commonly held belief. According to Rupert, it was commonly thought among North American chiropractors that one of the purposes of maintenance care is to "determine and treat subluxation" [6], confirmed in a similar study in Australia [7].

Judging from our results, there is no commonly agreed standard frequency of scheduled treatments but it would vary with the needs of the patient. This differs from the impression one gets when reading the case-report of one maintenance care patient by Wenban [8]. According to his report a pre-determined, hence probably rigid, treatment schedule was offered to the patient. We were unable to find other descriptions of this aspect in the literature but such a standardized approach appears unreasonable. How can one predict in advance the future clinical development and consequential treatment needs of an individual patient?

## Conclusion

The results of the present Danish study supported those of the previous Swedish study. There seems to be a common approach among different types of chiropractors, in relation to the management strategies for patients with low back pain. Maintenance care for low back pain appears to be used in order to prevent further events, in particular with patients who react well to treatment and who have a long history of previous problems. However, there is some conflicting evidence both in relation to whether improvement is necessary and as to whether maintenance care is used mainly to prevent further events or to treat patients with poorer outcome for a prolonged period of time. More information is needed on the indications and treatment program for maintenance care, before studying its clinical validity.

## Competing interests

The authors declare that they have no competing interests.

## Authors' contributions

LTM and MH collected and anlayzed the data and wrote the first draft, supervised by CLY. All authors read an approved of the final manuscript.

## Supplementary Material

Additional file 1 List of the nine different scenarios presented in the questionnaire used for the interview. Click here for file

Additional file 2 List of the seven choices (A-G) of management strategies, including a brief/colloquial description of each. Click here for file
