# Supplementary material for: The Nordic Maintenance Care Program – An interview study on the use of maintenance care in a selected group of Danish chiropractors
Source: Chiropr Osteopat. 2009 Jun 17;17:5. doi: 10.1186/1746-1340-17-5 (PMC2704232; doi:10.1186/1746-1340-17-5)
Supplement: Additional file 1 — List of the nine different scenarios presented in the questionnaire used for the interview. [file 1746-1340-17-5-S1.doc]

Additional file 1.

List of the nine different scenarios presented in the questionnaire used for the interview.

1. An acute attack of LBP of 2 days’ duration and no previous history of LBP. The pain is completely gone after 2 visits. The patient seems to be an uncomplicated person and capable to look after himself and his back.
2. An acute attack of LBP of 2 days’ duration and no previous history of LBP. The pain is completely gone after 2 visits. The patient is very worried that the pain will come back again. The patient asks if he could come back regularly to make sure this will not happen.
3. An acute attack of LBP of 2 days’ duration and no previous history of LBP. The pain is about 20% better after 6 visits.
4. An acute attack of LBP of 1 week’s duration. The patient has had several similar attacks over the past 12 months. The pain is completely gone after 2 weeks of treatment.
5. An acute attack of LBP of 1 week’s duration. The patient has had several similar attacks over the past 12 months, but the pain pattern has varied over the treatment and now, after six visits, the pain is 20% better.
6. The patient has had LBP intermittently over the past year. After he 2^nd^ visit, the pain was 50% better but today, after six visits there has been no further change.
7. The patient has had LBP intermittently over the past year. After 6 visits, the pain was 80% better, but after a further two treatments the last month, the problem has gradually got a bit worse.
8. The patient has had LBP intermittently over the past year. After the 2^nd^ visit the pain was 20% better, but today, after 6 visits over the past month, the patient has got gradually worse.
9. The patient has had LBP intermittently over the past year. After 6 visits the pain is 20% better. The symptoms come and go for no apparent reason. The patient appears tired and moody.
