# Supplementary material for: The Nordic Maintenance Care Program – An interview study on the use of maintenance care in a selected group of Danish chiropractors
Source: Chiropr Osteopat. 2009 Jun 17;17:5. doi: 10.1186/1746-1340-17-5 (PMC2704232; doi:10.1186/1746-1340-17-5)
Supplement: Additional file 2 — List of the seven choices (A-G) of management strategies, including a brief/colloquial description of each. [file 1746-1340-17-5-S2.doc]

Additional file 2.

List of the seven choices (A-G) of management strategies, including a brief/colloquial description of each.

- 1. I would refer the patient to another health care practitioner for a second opinion (“Second opinion”)
  2. I would tell the patient that the treatment is completed but that he is welcome to make a new appointment if the problem returns (“Quick fix”)
  3. I would not consider the treatment to be fully completed and would try a few more treatments and perhaps change my treatment strategy, until I am sure that I cannot do any more (“Try again”)
  4. I would advise the patient to seek additional treatment whilst following the patient (“External help – keep in touch”)
  5. I would follow this patient for a while, attempting to prolong the time period between visits until either the patient is asymptomatic or until we have found a suitable time lapse between check-ups to keep the patient symptom free (“Symptom-guided maintenance care”)
  6. I would recommend that the patient continues with regular visits regardless of symptoms, as long as clinical findings indicate treatment (e.g. spinal dysfunction/subluxation) (“Clinical findings-guided maintenance care”)
  7. Neither of the above. Please explain.
